# The possibilities and challenges of integrative medicine implementation in clinical psychology: a qualitative study in Indonesia

**DOI:** 10.1186/s12906-020-03019-x

**Published:** 2020-07-14

**Authors:** Andrian Liem

**Affiliations:** 1 Department of Psychology, University of Macau, Macao (SAR), China; 2 School of Psychology, The University of Queensland, Brisbane, Queensland Australia

**Keywords:** Complementary and alternative medicine, Integrative medicine, Holistic medicine, Mental health, Thematic analysis

## Abstract

**Background:**

Integrative medicine (IM), which is the integration of complementary and alternative medicine (CAM) into conventional health services, has been applied in some nations. Despite its highly relevant holistic approach with the improvement of mental health care using person-centred approach, there are limited studies that discuss IM, specifically in clinical psychology. Therefore, this qualitative study aimed to explore the perspectives of Indonesian clinical psychologists (CPs) on the possibilities and challenges of IM implementation in clinical psychology.

**Methods:**

Semi-structured interviews with 43 CPs who worked in public health centres were conducted between November 2016 and January 2017. A maximum variation sampling was used. Thematic analysis of interview transcripts was applied considering its flexibility to report and examine explicit and latent contents.

**Results:**

Three themes were identified from the analysis. First, the possibility of IM implementation in clinical psychology, which revealed two possible options that were centred on creating co-located services. Second, the challenges that covered (a) credibility, (b) acceptance, (c) procedure and facility, and (d) understanding and skill. Lastly, participants proposed four strategies to overcome these challenges, including: (a) certification, (b) facilities, (c) dialogue, and (d) regulations.

**Conclusion:**

Participants recognised the possibility of IM implementation in clinical psychology, particularly in clinical psychology services. This IM implementation may face challenges that could be overcome by dialogue between CPs and CAM practitioners as well as clear regulation from the government and professional psychology association.

## Background

Integrative medicine (IM) is defined as the integration of complementary and alternative medicine (CAM) into conventional medicine under holistic approach (or also known as CAM integration) aimed to achieve greater therapeutic effect [1, 2]. In IM, health is considered holisticly by valuing all aspects including physical, psychological, social, cultural, and spiritual beliefs [1–3]. This holistic approach was influenced by the philosophy of CAM that emphasizes the mind and body balance [4, 5]. In addition, CAM in some nations and cultures may be considered as and overlaped with traditional medicine because it is a part of the nation or culture’s own tradition [2, 6].

The World Mental Health Surveys in 25 nations found that the number of persons with mental disorder in high-income nations who reported visiting a CAM provider in the past year was double than in low-and-middle income nations [3]. Up to 18% of survey participants were combining conventional medicine and CAM treatments, which was predominant among persons with a severe disorder. Participants who integrated CAM into their mental health treatment reported relatively same level of satisfaction between these two modalities [3]. In addition, previous review found that education level did not affect people’s behaviour in combining CAM and conventional psychotherapy for treating mental disorders [1].

In medical settings, IM has been applied in some nations, for example, in the USA [4, 7], Australia [8, 9], China and India [1, 10], and European nations such as the UK, Sweden, and Denmark [9]. IM has also been applied for particular health services such as chronic disease management and palliative care [8, 9, 11]. The implementation of IM in these conventional health services followed various models or frameworks, and were dominated with general practitioners (GPs) as the gatekeepers [8, 9]. Nevertheless, the implementaion of IM in these nations and settings shared the same core understanding of an IM, which is the integration of CAM into conventional medicine.

### IM implementation in clinical psychology

The holistic approach of IM is highly relevant with the improvement of mental health care using person-centred approach [10]. The implementation of IM in clinical psychology will encourage clinical psychologists (CPs) to appreciate and treat their clients as a whole person [5]. For example, psychological treatment for clients can be customized according to the individual client’s needs and values, as well as acknowledgement of diverse cultures and beliefs [8]. In addition, the focus of mental health services with applied IM shifts from disorders treatment to prevention and wellbeing maintenance [1, 2].

Moreover, implementation of IM in clinical psychology may offer several advantages [2]. For example, IM may reduce the stigma of mental illness, because holistic approach lessens the body and mind dichotomy, which can lead to an increase of mental health services used by clients and their caregivers [1, 12]. IM may also be more cost-effective in preventing mental illnesses and reducing the demand on mental health treatments [2, 9]. Implementing IM in clinical psychology is also an alternative way to ease the burden of mental health professionals (HPs) shortage [1].

CPs should inform clients about all treatment options available, including CAM methods in IM services [4]. The information given must comprehensively cover the safety, efficacy/effectiveness and limitations of CAM methods to help clients make rational decision [13]. A survey among patients in primary care in the Netherlands found that good quality of information on CAM was needed because the participants aimed to combine it with conventional medicine to maximise the effect of both modalities [14]. These participants also reported that they wanted to make a shared decision with their GP about integrative treatment plan.

In order to discuss about CAM and provide accurate information about it in IM services, CPs need to have cultural sensitivity and knowledge on CAM methods and IM [4, 15]. Understanding of CAM methods is also an important factor for CPs to perceive CAM positively and increase communication effectivity and trust with clients [16, 17]. Surveys in 25 nations found that two-thirds of people with mental disorder did not disclose their CAM use to their conventional health practitioners due to fear of disapproval and uncertainty about their health practitioners’ ability to integrate CAM [3]. Studies in African nations and Australia also found that lack of CAM understanding among conventional HPs may affect their perspective on IM and willingness to collaborate with CAM practitioners (CAMPs) [15, 18].

Information about CAM and IM has been integrated into conventional health education, mainly in medicine, nursing, and pharmacy education programs [19, 20]. However, the inclusion of CAM in these conventional health education curriculums varied between schools and institutions, resulting in inconsistent understanding of CAM and mixed attitudes towards IM [5, 15]. Additionally, inconsistent CAM understanding may lead to diverse implementation of IM and potential challenges for certifying the service [19]. While in psychology education, especially in Asian nations, the absence of CAM information may be the result of imported psychology curricula from Euro-American education, which put emphasis on conventional psychotherapy [21, 22].

### The current study

Although IM has been broadly applied in health services, which mostly in high-income nations, there are limited studies which discuss IM specifically in clinical psychology. Implementing IM in clinical psychology is needed in Asian nations because not all western conventional psychotherapy methods are suitable for Asian people [23, 24]. IM implementation in clinical psychology may also address the shortage of mental HPs and services in Indonesia (i.e. the availability of psychiatrist and psychologist are 3 and 1 per 1,000,000 people, respectively) and reduce stigma towards mental disorders including feelings of shame in seeking help for mental disorders [16, 25].

Similar to CPs in high income nations where IM has been implemented, Indonesian CPs also need to prepare themselves for CAM discussion with their clients because IM practice has been regulated by the Ministry of Health [26] that imply CPs not to prohibit but should educate their clients about CAM if they are interested to use it. Moreover, CAM is part Indonesian people daily lives [6, 11]. In a 2018 national survey, about 65% Indonesians have used CAM treatments categorised as treatments using skill (*keterampilan manual*) with or without instrument such as message, bone setter, and acupuncture [27]. Additionally, a spiritual and religious-based CAM treatment were also commonly used by Indonesian people since religion is part of Indonesian culture [11]. Indonesian CPs might support this CAM use through applying IM, as previous studies have presented evidence for CAM effectiveness, for example, in promoting quality of life and reducing some health issues such as insomnia and back pain [4, 13]. It is important to note that there is an abundance of literature about CAM classifications and efficacy/effectiveness (e.g. 4, 5), but this topic is beyond the scope of this qualitative study and will not be discussed here.

This current exploratory study was part of a mixed-methods research on CAM among Indonesian CPs. The participants in the first phase, a national survey, showed lack of CAM knowledge and positive attitude towards CAM, and reported a need of CAM education [16, 24]. However, this quantitative survey did not investigate further about the potentials and challenges for integrating CAM into clinical psychology. Therefore, the current qualitative study aimed to fill this gap by exploring Indonesian CPs’ perspectives on possibilities and challenges of IM implementation in clinical psychology.

## Methods

### Research design

This study used qualitative design based on constructivist epistemology approach. This epistemology approach was chosen because of its purpose to explore what is assumed to be socially constructed dynamic reality [28], which in this qualitative study was Indonesian CPs‘perspective on possibilities and challenges of IM implementation in clinical psychology. In the constructivist epistemology approach, the researcher’s and participant’s interests are entangled in the interpretation process which does not create absolute objectivity [29]. A disclosure of researcher’s personal view as a reflexivity process is important in maintaining the study’s trustworthiness in this epistemology approach. The researcher reflected that he has a positive attitude towards implementation of IM in clinical psychology due to his accumulated knowledge and experience on CAM. However, the researcher is concerned about unregulated use of CAM treatments in clinical psychology services that may endanger the CP and his/her client into a malpractice. This is the main motivation for the researcher to conduct a mixed-method study on CAM among CPs in Indonesia.

### Sampling and participants

A maximum variation sampling [30] was used in this study to achieve a greater understanding of possibilities and challenges of IM implementation in clinical psychology. Therefore, CPs who worked at 43 public health centres (PHCs) in Special Region of Yogyakarta province, Indonesia, were selected. These 43 PHCs were selected because the inclusion of clinical psychology services at PHCs in Indonesia was initiated in these PHCs in 2004 [25]. Participants ranged from 25 to 42 years old and were originally from Java, Sumatra, and Kalimantan Island. They had been practicing as psychologists for 10 months to 18 years (see Supplement A). There was only one male participant, therefore, ‘she’ is used to discuss all interview responses in this study to maintain participants’ anonymity.

### Data collection

This study, as a part of mixed-methods research on CAM among Indonesian CPs, had been granted ethics approval by the Ethics Committee of the School of Psychology at the University of Queensland (approval number: 16-PSYCH-PHD-08-JH). Prior to data collection, research permission from the Indonesian Clinical Psychologist Association (IPK) was sought, which was granted after presenting the research proposal to its executive members. After the first phase of mixed-methods research, the author mailed an introduction letter, along with an information sheet and research permission letter from the IPK, to the potential participants in 43 PHCs whose postal addresses were publicly available. The introduction letter explained the aim of the study and asked participant’s willingness to be interviewed. Participants were also informed that they may choose not to participate without any consequences.

None of the potential participants declined the interview requests, so the author contacted them directly through email address and phone number provided by the IPK to arrange interview time. All participants were interviewed by the author, who is a male, PhD candidate, and also an Indonesian clinical psychologist with skills in collecting qualitative data, between November 2016 and January 2017. Prior to the face-to-face interview and signing the consent form, all participants were given the chance to ask questions related to the research. All participants voluntarily agreed to be interviewed at their suggested time and place. The interviews lasted between 30 and 100 min and were audio-recorded. Participants received a compensation of Rp 100,000 (equal to USD 7).

Semi-structured interviews were used to collect data, assisted by an interview schedule. Process of the interview schedule development and the pilot interviews results had been reported elsewhere [31]. In the interview, participants were asked about their perspectives on: (a) the possibilities of IM implementation in clinical psychology and (b) the challenges of IM implementation in clinical psychology, including strategies to overcome these challenges.

### Data analysis

All interviews were transcribed, assisted by a research assistant (RA), to begin analysis. The author transcribed the first five interviews which were given to the RA as examples for transcription quality standards. The RA transcribed the sixth through tenth interviews which were evaluated by the author before transcribing the rest of interview recordings. Only minor adjustments were needed in regarding incorrect medical terms and anonymizing participant’s name or other’s names. The RA continued to transcribe the remaining interview recordings and all transcripts were reviewed by the author for its accuracy. These double-checking practices were conducted to improve research credibility and accuracy of data interpretation [32]. Although the transcripts were not returned to participants, credibility was maintained by confirming what the interviewer (the author) had understood during the interview process [32]. As this study aimed to achieve a greater understanding of the explored issues by employing a maximum variation, data saturation was not prioritised, but it was noted that the data were saturated during the middle of serial interviews with 43 participants.

Thematic analysis was used to analyse the interviews transcripts due to its flexibility to both report and examine explicit and latent contents [33]. This study followed steps of thematic analysis from previous studies [34, 35] that cover: (a) initial coding, (b) searching for themes, and (c) analysis. The initial coding step was conducted manually using word and colour on a word processor by the author. Subsequently, potential sub-themes and themes were generated from re-organising the initial code based on its proximity. These sub-themes and themes were then analysed by consulting a senior lecturer in Indonesian language and culture who has expertise in qualitative methodology, to improve analysis and interpretation conformability [32]. This study was reported following the consolidated criteria for reporting qualitative research (COREQ) checklist [36] to improve its clarity and quality.

## Results

Participant’s number is used in brackets to represent extracts and quotes. For example, ‘(P34)’ represents a quote from ‘Participant 34’. The results are organised into three themes, according to the responses from the participants. The first was the participants’ thought of how IM could be implemented in clinical psychology, which revealed two possible scenarios for integrating CAM into clinical practice given by the participants. The next two themes were the challenges of IM implementation that might occur, followed by strategies that might be applied to overcome these challenges.

### The possibilities of IM implementation in clinical psychology

**Fig. 1:**
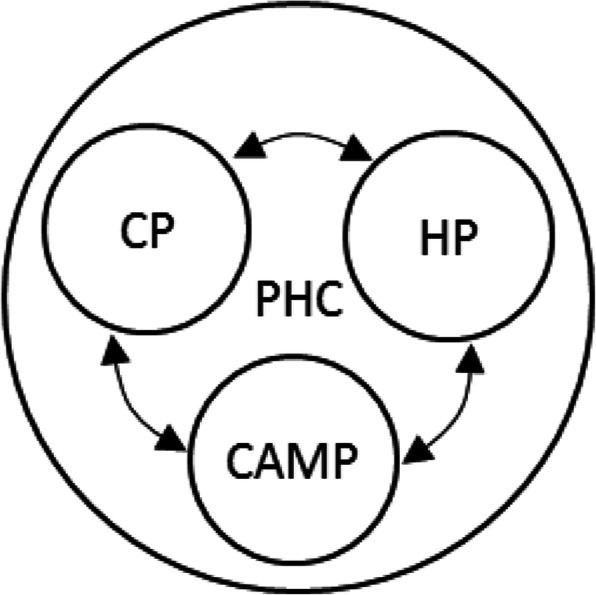
The first possibility for IM implementation in clinical psychology. *Note*. PHC = Public Health Centre; CP = Clinical psychologist; HP = Health professional; CAMP = CAM practitioner; ←→ = Referral.

When asked about how participants would envision the implementation of IM in clinical psychology, they offered two possible options, particularly in clinical psychology services that cover all activities to assist an individual and/or group aimed to prevent and solve psychological problems [37]. The first possibility (Fig. 1) is CPs are only to give recommendations and referrals, because CPs already have excessive responsibilities and providing CAM themselves would create an additional burden. The majority of participants endorsed co-located settings for practice locations where conventional HPs and CAMPs were placed together and worked collaboratively at the PHCs. The primary advantages of this scenario were time efficiency and effective communication. For example, Participant 22 explained that CPs have limited time in one session. Therefore, having CAMPs at the same place would save CPs’ and clients’ time when a referral is needed. In addition, CPs, CAMPs, and other HPs would be able to discuss and plan holistic treatment plans together.

**Fig. 2:**
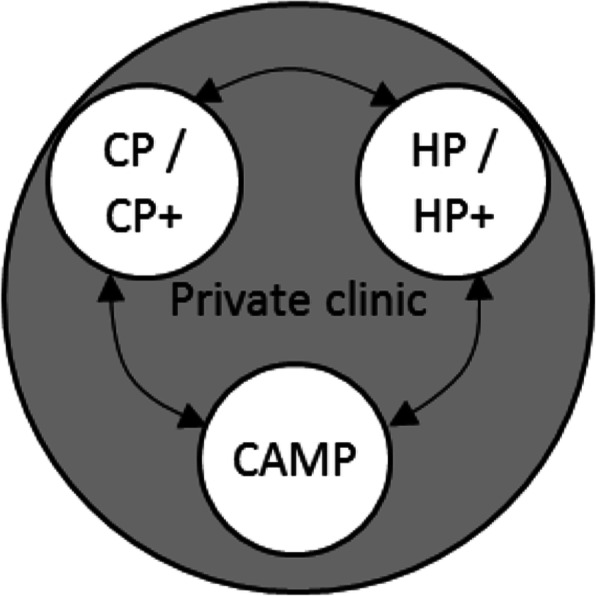
The extension of first possibility for IM implementation in clinical psychology. *Note*. CP = Clinical psychologist; CP+ = Clinical psychologist with CAM skills; HP = Health professional; HP+ = Health professional with CAM skills; CAMP = CAM practitioner; ←→ = Referral.

The first possibility proposed by participants was extended to IM implementation at private clinics where CPs might work together with CAMPs (see Fig. 2 below), as they realised that CPs may work at private clinics and have a different situation from those in the PHC system. Participant 37 added that CPs with CAM skills who work at PHCs should not use their skills there, but could possibly practice them at private clinics and work as an associate (denoted in Fig. 2 by CP*+*). Also, some participants offered a perspective that conventional health services should have joint agreements with credible CAMPs in remote areas so that clients can be referred to them. This idea would be particularly useful for clients in rural and remote areas. This is because of the high rate of early-termination among rural clients due to the geographical difficulty of visiting conventional health services, and thus many go to CAMPs instead. However, although collaborative work is encouraged in this proposed model, participants emphasised that the CAMPs’ position would be subordinate to conventional health practitioners. Additionally, CAM treatments practiced by either CAMP or CP+ could be used only as an add-on or after conventional medicine is given:

“ … it must be different between pure acupuncturists and CPs with acupuncture skill, for example, the treatment of how to make a rapport with client, etc. Psychotherapy must be as the primary intervention to differentiate them [CPs+] from the acupuncturist.” (P34).

**Fig. 3:**
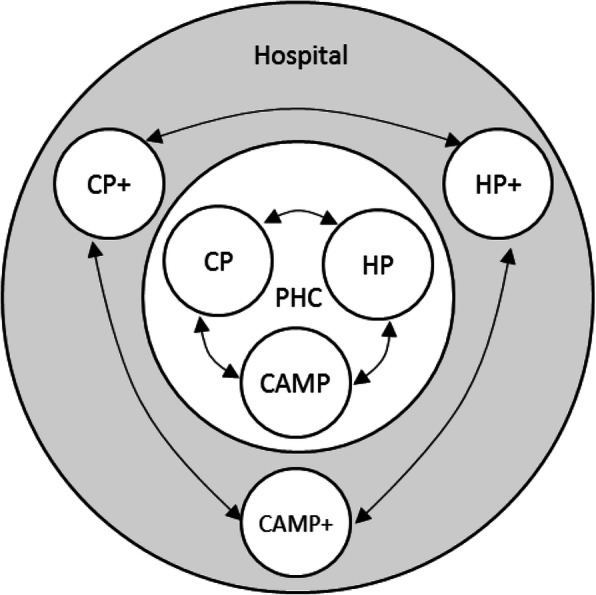
The second possibility for IM implementation in clinical psychology. *Note*. PHC = Public Health Centre; CP = Clinical psychologist; CP+ = Clinical psychologist with CAM skills; HP = Health professional; HP+ = Health professional with CAM skills; CAMP = CAM practitioner; CAMP+ = CAMP with higher skills and experience and more complicated CAM treatment; ←→ = Referral.

The second suggested possibility was co-location using a multi-tiered system. In this suggestion, CPs and HPs at the PHCs would only provide basic clinical services, while CPs and HPs at the hospital may include CAM treatments in their practice (denoted in Fig. 3 by CP+ and HP*+*). As justified by Participant 37, CPs at PHCs should deliver only the essential psychological services, for example, assessments and mild mental disorder interventions, as general practitioners (GPs) would do. As a consequence, more complex services should be referred to CP+ at the hospital who would be expected to have better skills and facilities. Participants advised that one CP+ should specialise only in one particular CAM treatment, like medical specialists do.

On the one hand, most participants agreed that CAM treatments provided by CP+ should have a connection with psychological intervention, for example, spiritual-religious therapy (SRT), music therapy, aromatherapy, yoga, and meditation. On the other hand, participants expressed their resistance towards acupuncture, herbal medicine, and dietary-supplements therapy because of the perceived high risk of harm to clients. A dissenting opinion emerged for acupressure, as some participants supported CP+ to provide it but others disagreed. However, participants also highlighted that CP+ may provide any CAM treatment as long as they were certified, and the CAM treatment is supported by scientific evidence and regulated by the government and professional associations. In addition, CAMPs who are co-located at PHCs should only provide CAM treatment that is familiar to the general public, such as herbal medicine. CAMPs with more skills and experience, and more complicated CAM treatment practices were advised to be co-located at hospitals together with CP+ and HP+.

### Challenges for IM implementation in clinical psychology

Participants discussed the challenges of IM implementation in clinical psychology, particularly in clinical psychology services, which covered the following sub-themes: (a) credibility, (b) acceptance, (c) procedure and facility, and (d) understanding and skill. In addition, they discussed challenges which may come from four sources: (a) clients/general people, (b) internal CP’s community, (c) other conventional HPs, (d) CAM practitioners. This sub-section discusses these challenges by incorporating the four sub-themes and four sources together.

The first sub-theme, credibility, was highlighted by participants. CPs who recommend, refer, or provide CAM treatments may generate misconceptions about the psychology profession among clients/general people. One participant summarised:

… the profession of psychology itself has not been searched for and known [by Indonesian people] … about what CPs can help, and [for many people this] is still unclear. Let alone if CPs refer clients to a CAM practitioner or give CAM [in their practice]; it will make people misunderstand about the psychology profession. (P18).

In addition, some participants expressed their worries that IM implementation in clinical psychology would damage the professional image of psychologists. Credibility challenges may also come from the internal CP’s community in the form of professional ambiguity because CPs who provide CAM in their practice might be questioned in their work as psychologists or CAM practitioners. In addition, there is no certification process for CAM competency from a professional organisation. Participants also voiced their concern about CAM practitioners’ credibility and potential legal issues, particularly in regard to training from trustworthy CAM institutions as well as the effectiveness and safety of CAM treatments used by CAM practitioners.

The second challenge was acceptance, whereby participants perceived that clients may hesitate or reject IM. Moreover, some participants assumed that IM implementation in clinical psychology might increase the risk of complaints from clients, especially regarding CAM treatments that involve direct physical contact. Colleagues and senior psychologists also showed their hesitation towards IM and negatively perceived the integration because they believed that psychologists should only use conventional psychotherapy intervention. For example:

“Psychology takes a long time to open up and accept new therapy techniques. [While] CAM is still unfamiliar and has little supporting scientific evidence, so the psychology community still finds it difficult to accept, let alone if we want [CAM] to be integrated [into conventional psychotherapy].” (P23).

Participants also doubted that IM implementation would be accepted by other HPs because CAM itself is still being debated among other HP communities. In addition, participants worried that if CPs provided the same treatments as other HPs, this may cause potential conflicts because other HPs may interpret that CPs are trying to ‘steal their jobs’. For example, a CP who practices dietary-supplement consultation or acupuncture might be perceived negatively by nutritionists, or physicians who provide acupuncture. Participants were also concerned that CAMPs may also have the same perception about CPs who attempt to integrate CAM into their services, for example, a CP who teaches yoga to their clients may be disliked by yoga trainers because they might be perceived as a competitor.

CAM understanding and skills was the third challenge for IM. Participants were concerned about the diverse understanding of CAM in the CP’s community due to the lack of CAM knowledge and varied information that CPs had. Moreover, participants were aware that some of their colleagues had been practicing some CAM treatments as part of psychological interventions, for example, SRT and acupressure, but participants questioned these actions since the colleagues’ CAM competencies were unclear. Participants underlined that insufficient CAM understanding also resulted in difficulty in communicating and educating clients about CAM, as exemplified: “… because we also must explain in detail to clients so they can decide. Including to our colleagues and other health professions so they do not misunderstand. But it is difficult [to explain] if we do not [ourselves] understand [about CAM].” (P40). CPs who integrate CAM also face challenge in ensuring that conventional psychotherapy is still used as the primary technique and their CAM service is different from that provided by CAMPs or other HPs who also provide CAM.

The last challenge was about procedure and facilities, especially for CPs who work at PHCs. The IM implementation in clinical psychology services might improve psychological intervention. However, participants worried that clients might terminate their sessions early when they ‘feel better’ and rather depend more on CAM. Under a co-located, multi-tiered system scenario, some participants were concerned that clients may not comply when being referred to hospitals to meet CP+ or HP+ due to time and geographical difficulties. Moreover, participants expressed that limited time for each session and the facilities at conventional health services may be a challenge when CAM is integrated by CPs. The bureaucracy at conventional health centres also hinders participants in getting permission for taking CAM workshops during weekdays. Participants said the current system do not allow them to refer clients to HPs + or CAMPs because these referrals should be made by a physician. Moreover, participants felt that the psychology profession was treated unequally by the Health Ministry in comparison to other HPs, so CPs may not have the power to integrate CAM into their services. For example, as expressed by Participant 22, “At the Health Department we are still considered second class when compared with others [health professions]. Consequently, there are a lot of gaps, financially as well as in terms of our authority.” Participants also underlined the need to implement coordination procedure between conventional HPs and CAMPs because, at present, there are no such procedure.

### Strategies to implement IM in clinical psychology

Four strategies were proposed by participants to address the challenges of implementing IM in psychology services: (a) certification, (b) facilities, (c) dialogue, and (d) regulations. Certification was primarily proposed for CAM practitioners (CAMPs) in order to increase their credibility and create a more positive image of CAM treatments. Participants also underlined that professional associations should conduct competency or credential exams for CPs who want to implement IM to guard against malpractice. Certification among CPs was also perceived as an alternative to minimise the risk of conflict with CAMPs because CPs would provide CAM as an add-on to conventional psychotherapy which distinguish them from CAMPs.

Improving facilities at conventional health services was suggested if IM was to be implemented in clinical psychology. For example, more convenient therapy rooms would be needed if CPs wanted to conduct yoga and meditation sessions. Another facility that may be required is computers with internet access to assist CP’s in providing education to clients about CAM before making recommendations or referrals, or for clients case conference purpose with other HPs and CAMPs.

The third critical strategy advised by participants for IM implementation was dialogue between CPs, CAMPs, and other HPs. Since CAMPs have diverse training backgrounds, participants suggested that CPs need to explain about conventional mental health interventions before the two professions could work together. This step is crucial to ensure CAMPs have the same perspective with CPs and avoid carrying out overlapping or contradictory interventions when they work collaboratively. Dialogue with other HPs was also described as fundamental when conducting IM. Therefore, participants encouraged CPs, HPs, and CAMPs to have case conferences when planning a treatment and discussing clients’ progress. Additionally, dialogue was also needed within the internal psychology community. Hence, participants urged that CPs establish a CAM interest group as exemplified by one participant: “For instance, CPs who provide CAM could create a ‘complementary psychologist association’. So, the members are CPs with expertise in CAM and I can refer [clients] to the members and I can also learn from them.” (P37).

**Fig. 4:**
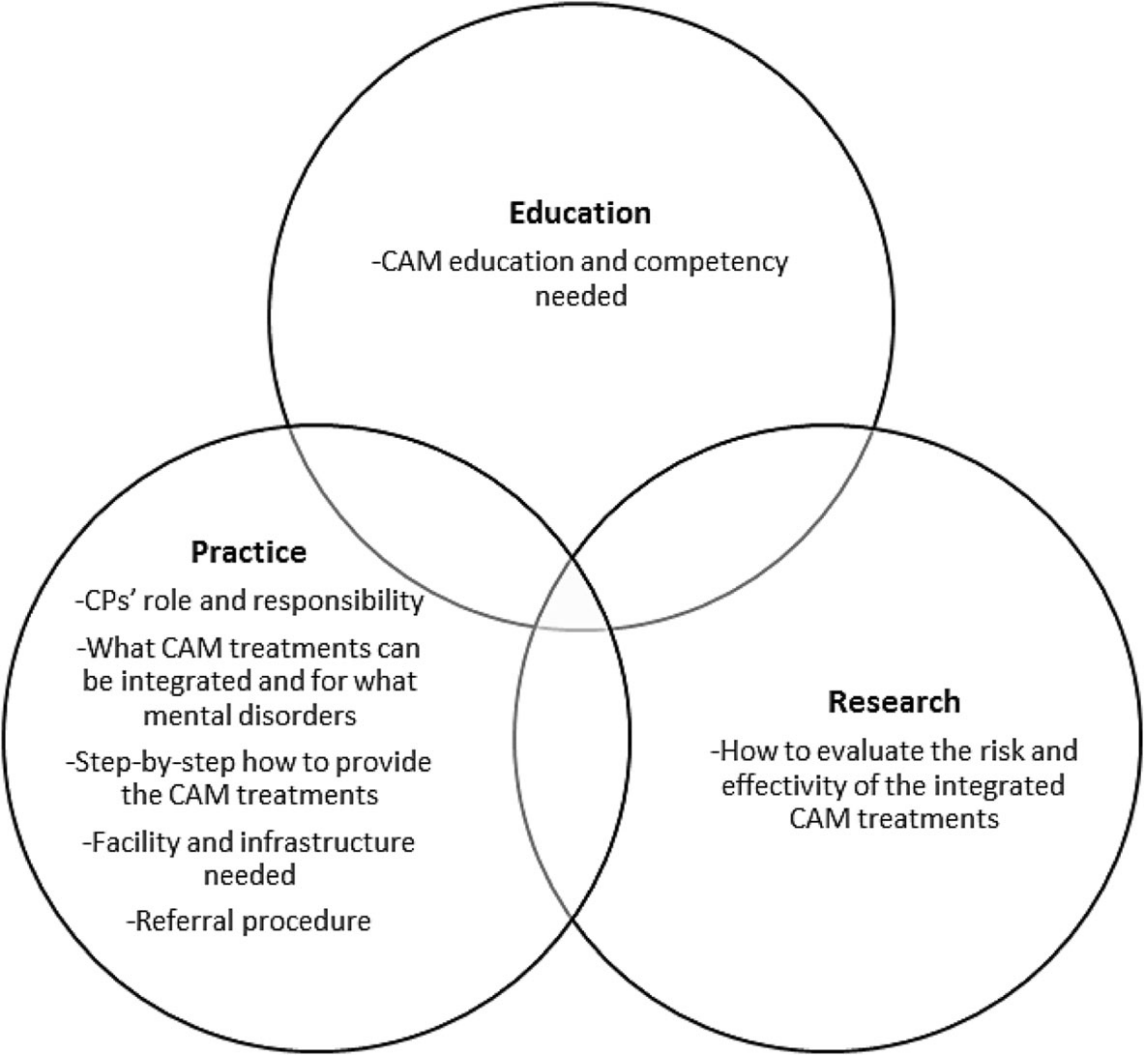
Proposed standard operating procedure for IM implementation in clinical psychology

Lastly, the participants proposed the creation of regulations, particularly from the government and professional associations, for implementing IM. Such regulation is important because, for example, it may prevent misconceptions or negative perceptions about psychology professionals among clients or the general public and other HPs, for example, “This [regulation] can prevent our profession [CP] from being equated with *dukun* [shaman] and make people not misunderstand [about psychologist profession].” (P40). There were three regulations outlined by participants. First, to create a standard operating procedure (SOP) that should cover three areas and is summarised in Fig. 4. The second proposed IM regulation was the standardisation of CAM competency and a certification examination process. The last proposed regulation was about how to collaborate with CAMP, particularly regarding CAM referrals that would help CP+ and CAMP to work collaboratively without crossing their scope of practices and minimise any liability issues.

## Discussion

This qualitative interview study explored the possibilities and challenges of IM implementation in clinical psychology in Indonesia from the perspective of CPs. The participants proposed two possible options of IM implementation in clinical psychology, which were centred on creating co-located settings in clinical psychology services. The main reasons were the participants’ concerns about the limited time in each session and potential additions to their work burden. In addition, participants believed that co-located settings would allow conventional HPs and CAMPs to work collaboratively. These possibilities to implement IM in clinical psychology may also help to reduce stigma towards mental healthcare as IM encourages a holistic approach [1, 38]. Moreover, co-located settings for IM would assist clients to avoid untrustworthy and less credible CAMPs [1, 39]. These proposed possibilities might also encourage clients to disclose their use of CAM to their physician and psychologist so that potential health risks (i.e. adverse effects) could be avoided [5].

Moreover, previous studies of successful IM services in Canada showed that co-located settings allowed physicians to learn from CAMPs, and vice versa, so they could work effectively with fewer misunderstandings [7]. CPs working in co-located settings for IM may also learn from CAMPs about CAM treatments related to mental health problems, and CAMPs may gain greater knowledge about conventional psychotherapy, so mutual understandings can be promoted [38, 40]. IM services in co-located settings may also be empowered to act as internship places for provisional CPs to learn about CAM and encourage them to apply IM in their future careers [13]. This has been demonstrated among nursing, pharmacy, and medical degree students in Hong Kong [41].

The possibility of multi-tiered system advised by participants is in line with previous studies that have endorsed CAM at PHCs because of their similar views on holistic health, health promotion, and disease prevention [9, 42]. Moreover, IM implementation at PHCs may reduce hospital referrals and enhance clients’ quality of life [43]. Participants in this study recommended clinical psychologists with CAM skills (CPs+) to only integrate CAM treatments that are related to conventional psychology intervention which mostly are classified as MBTs (i.e. meditation). Despite the low risk of acupressure when integrated into psychological services [40], some participants resisted using CAM treatments which involve direct physical contact, which was a similar finding among CPs in the USA in a previous study; CAM involving physical contact was seen to be a boundary violation and CPs were discouraged from practicing such treatments [13].

Participants perceived that the main challenge for implementing IM in clinical psychology was the issue of credibility, which was also found in a previous study among CPs in the USA [13]. In addition, distrust of CAMPs and CAM treatments may affect insurance policies, whereby the insurance company may not cover CAM treatments [38]. Even if the CP+ has some competency in a particular CAM treatment, the next challenge that may arise is dual accreditations, as both a psychologist and a CAM practitioner; such licencing or accreditation has yet to be developed or regulated by professional psychology associations to avoid confusion [39]. In line with previous studies in Canada and the USA [9, 43], concerns over credibility were also related to the credentials held by CAMPs, which may be inconsistent, due to the diverse training and licensing of CAM. The lack of CAMPs’ credentials leads to scepticism about the efficacy, effectiveness, and safety of CAM, as found in the current study and also expressed by American CPs [39].

The current qualitative study found that certification of CAM practice, either by CPs or CAMPs, may be used as a strategy to increase the credibility of, and minimise potential conflicts between CPs + and CAMPs. The Indonesian regulation of CAM practice at conventional health services also maintains that HPs + and CAMPs must be certified for the CAM treatment they provide and that practitioners must be endorsed by the related professional associations [26]. However, the psychology community in Indonesia at present does not have a relevant interest group for CAM, which has existed amongst Australian psychologists [44]. Consequently, it is difficult for Indonesian CPs to gain credentials in a particular CAM treatment. A previous study showed that a CAM interest group among physicians in the USA, named the American Holistic Medical Association, enhanced positive attitudes towards CAM in general and towards IM particularly [38]. Alternatively, professional associations or IM institutions may organise informal gatherings like luncheons as performed by successful IM services in Hong Kong [41]. In addition, national-standard certification for CAMPs, for example as shown in Australia which is the first nation to register traditional Chinese medicine practitioners [20], may improve their credibility.

Participants advised for a dialogue to happen between CPs and CAMPs as a strategy to implement IM, an advice which was also expressed by CPs in the USA [39] and identified as one of the critical factors in running effective IM in Hong Kong [41] and Canada [7]. Dialogue in professional settings was exemplified through case conferences involving HPs and CAMPs. However, this multidisciplinary case conference may only happen if trust between HPs and CAMPS has already been built [7]. Participants’ recommendation of improving facilities such as providing computers with internet access was also discovered in previous studies in the USA [38] and Australia [9]. The technology can be used to effectively record and restrict what information about clients can be shared for multidisciplinary case conferences.

Regarding the regulation to implement IM in clinical psychology, the psychology professional association need to resolve the absence of policy for CPs who integrate CAM into their practice. The current Indonesian psychology code of ethics [37] and the clinical psychology services’ standards [45] do not regulate dual licensing for CPs as both a clinical psychologist and a CAM practitioner, and this is necessary in order for CPs to have clear guidelines for including CAM in their clinical practice. This finding is similar with studies among American and Australian psychologists [39, 44] where they were uncertain about the scope of practice of CPs who also had a license as a CAMP, for example, as an acupuncturist. However, the current qualitative study discovered that participants perceived that psychology professional associations do not perform their regulatory functions to the best possible effect.

The psychology code of ethics in Indonesia dictates that psychologists, when they make professional decisions, must rely on scientific knowledge that has been scientifically tested and accepted within the psychology community [37]. However, this clause might be interpreted in diverse ways when it comes to IM implementation in clinical psychology, as was found in a previous study amongst American psychologists who were uncertain of how to classify CAM research as either a scientific knowledge or pseudoscience [39]. A critique of CAM research, particularly on the rigorousness of their methodology, has been highlighted previously by the medical community [4, 43].

Conversely, it has been argued that double-blind randomised controlled trials (RCT), as the gold standard of medical research, need to be adjusted for CAM research [20, 38, 39]. The primary reason is that many CAM treatments depend on the CAMP’s skills and it is difficult to do double-blind procedures. For example, acupuncturists can identify if they are assigned with active or placebo acupuncture needles, so double-blinding has been challenging [46]. In addition, the relationship between a client and CAMP is also essential for a holistic approach in CAM, so assigning clients and CAMPs into double-blind RCT is not a straight-forward process. Psychologists were encouraged to be proactively involved in CAM research to assess the likelihood of implementing IM in clinical psychology [13]. Despite extremely limited CAM studies in psychology, CPs need to remember that the lack of research does not mean that CAM is not useful [7, 13].

Participants advised that the regulation for IM implementation should also be incorporated as Continuing Professional Development (CPD). This strategy may be implemented through a new regulation for clinical psychology licensing and services [47] where CPs must collect a certain number of CPD points within a particular period in order to renew their license. IM services in Hong Kong successfully applied this regulation for nurses, pharmacists, and physicians at their institutions by periodically inviting keynote speakers to give seminars on relevant topics [41]. Government and professional associations also need to regulate internships and research of IM services at co-located settings. Preceding studies have shown that clear regulations on IM implementation supported the enhancement of novice HPs’ CAM understanding and skills during internships, and encouraged them to do CAM research in the future [4, 9, 13].

### Limitations and recommendations

Although participants in this novel study provided a comprehensive view of the possibilities and challenges of IM implementation in clinical psychology, there were two major limitations that need to be considered. First, all interviews were conducted among CPs who worked at PHCs. Thus, the results might not accurately represent CPs in different health service settings. Future study may investigate perspective of CPs in hospitals and private clinics. Second, it was discovered that the role of psychology professional associations was perceived very important in implementing IM in clinical psychology. However, only two participants in this study were actively involved in the association as executive members, which their colleagues may have different opinions. Therefore, future research is needed to explore other executive members’ perspective. Perspective of government officials (i.e. those from the Health Ministry) on IM, particularly on ethics and regulations of IM implementation, is also interesting to be explored in future study.

## Conclusion

Indonesian CPs in this qualitative study recognised the possibilities of IM implementation in clinical psychology that were centred on creating co-located settings, particularly in clinical psychology services. However, this IM implementation may face multiple challenges such as acceptance and credibility issues. Therefore, a dialogue between CPs and CAM practitioners was strongly advised as one of the proposed strategies to overcome the challenges of implementing IM in clinical psychology; and supported by comprehensive and substantial regulations, both from the government and professional association.

## Supplementary information

**Additional file 1.**

**Additional file 2.**

## Data Availability

The data that support the findings of this study are not publicly available due to risks to participants but available from the corresponding author upon reasonable request.

## Abbreviations

CAM: Complementary and alternative medicine
CAMP: CAM practitioner
CAMP+: CAMP with higher skills and experience and more complicated CAM treatment
COREQ: Consolidated criteria for reporting qualitative research
CP: Clinical psychologist
CP+: Clinical psychologist with CAM skill
CPD: Continuing Professional Development
GP: General practitioner
HP: Health professional
HP+: Health professional with CAM skill
IM: Integrative medicine
IPK: Indonesian Clinical Psychologist Association (*Ikatan Psikolog Klinis*)
PHC: Public health centres
RA: Research assistant
RCT: Randomised controlled trials
