# Supplementary material for: The possibilities and challenges of integrative medicine implementation in clinical psychology: a qualitative study in Indonesia
Source: BMC Complement Med Ther. 2020 Jul 14;20:223. doi: 10.1186/s12906-020-03019-x (PMC7362562; doi:10.1186/s12906-020-03019-x)
Supplement: Supplementary file 1 — Additional file 1. [file 12906_2020_3019_MOESM1_ESM.docx]

# Supplement A

Participants’ age and years of practice

| **Participant** | **Age** | **Level (based on years of practice)** |
| --- | --- | --- |
| 1 | 31-35 | Middle |
| 2 | ≤30 | Novice |
| 3 | ≤30 | Novice |
| 4 | 31-35 | Junior |
| 5 | 31-35 | Novice |
| 6 | ≥41 | Senior |
| 7 | 36-40 | Middle |
| 8 | ≤30 | Novice |
| 9 | ≤30 | Novice |
| 10 | 31-35 | Junior |
| 11 | ≤30 | Novice |
| 12 | 36-40 | Senior |
| 13 | 31-35 | Junior |
| 14 | ≤30 | Junior |
| 15 | ≤30 | Novice |
| 16 | ≤30 | Novice |
| 17 | ≤30 | Junior |
| 18 | ≤30 | Novice |
| 19 | ≤30 | Middle |
| 20 | 31-35 | Junior |
| 21 | 31-35 | Junior |
| 22 | ≥41 | Senior |
| 23 | 36-40 | Senior |
| 24 | 36-40 | Senior |
| 25 | 31-35 | Middle |
| 26 | ≤30 | Novice |
| 27 | 36-40 | Senior |
| 28 | 31-35 | Middle |
| 29 | 36-40 | Senior |
| 30 | ≤30 | Novice |
| 31 | 31-35 | Novice |
| 32 | 36-40 | Middle |
| 33 | 36-40 | Senior |
| 34 | 36-40 | Middle |
| 35 | 31-35 | Junior |
| 36 | 31-35 | Junior |
| 37 | 31-35 | Middle |
| 38 | ≤30 | Middle |
| 39 | 36-40 | Senior |
| 40 | ≥41 | Senior |
| 41 | 31-35 | Senior |
| 42 | 36-40 | Junior |
| 43 | 36-40 | Middle |

*Note*. Novice-level = ≤2.0 years; Junior-level = 2.1-4.0 years; Middle-level = 4.1-8.0 years; Senior-level = ≥8.1 years. The exact age and years of practice were covered to protect participant’s anonymity.
